# Late diagnosis of SLC34A3-related hereditary hypophosphatemic osteomalacia following a low-energy proximal humeral 4-part fracture

**DOI:** 10.1093/jbmrpl/ziag137

**Published:** 2026-08-19

**Authors:** Toru Morimoto

**Affiliations:** Department of Orthopaedic Surgery, Kochi Prefectural Hata Kenmin Hospital, Sukumo, Kochi 788-0785, Japan

**Keywords:** hereditary hypophosphatemic rickets with hypercalciuria (HHRH), *SLC34A3,* renal phosphate wasting, hypophosphatemic osteomalacia, fragility fracture, proximal humeral fracture, late-onset diagnosis, serum phosphate

## Abstract

Hereditary hypophosphatemic rickets with hypercalciuria (HHRH) is a rare FGF23-independent renal phosphate-wasting disorder caused by biallelic variants in *SLC34A3*, typically diagnosed in childhood or early adulthood. Delayed recognition in elderly individuals is uncommon, and the clinical features leading to diagnosis in this population remain poorly characterized. We report a case of a 77-yr-old Japanese man with HHRH who remained undiagnosed until advanced age and was identified following a proximal humeral fracture sustained after a low-energy fall. The severity of the fracture, together with his history of multiple previous fractures, prompted biochemical evaluation for secondary causes of skeletal fragility. Biochemical assessment revealed hypophosphatemia with preserved renal function, elevated 1,25(OH)2D3 levels, hypercalciuria, and reduced renal phosphate reabsorption. Intact FGF23 was mildly above the assay reference limit and was not appropriately suppressed in the setting of hypophosphatemia. BMD was in the osteoporotic range at the FN, but it did not distinguish osteoporosis from an underlying mineralization disorder. Genetic analysis identified a homozygous synonymous variant in *SLC34A3* (c.942G > C, p.Ala314=) in the context of a characteristic biochemical phenotype consistent with HHRH. Oral phosphate supplementation alone resulted in rapid normalization of serum phosphate levels, without deterioration in renal function or worsening hypercalciuria. This case suggests that *SLC34A3*-related phosphate-wasting disorders may remain clinically unrecognized until late adulthood. Importantly, it highlights the value of biochemical evaluation, including the measurement of serum phosphate and the assessment of renal phosphate handling, in patients with otherwise unexplained skeletal fragility. Phosphate-wasting disorders should be considered in patients with recurrent fractures or skeletal fragility that is not fully explained by BMD or the reported injury mechanism.

## Graphical Abstract

**Graphical Abstract:**
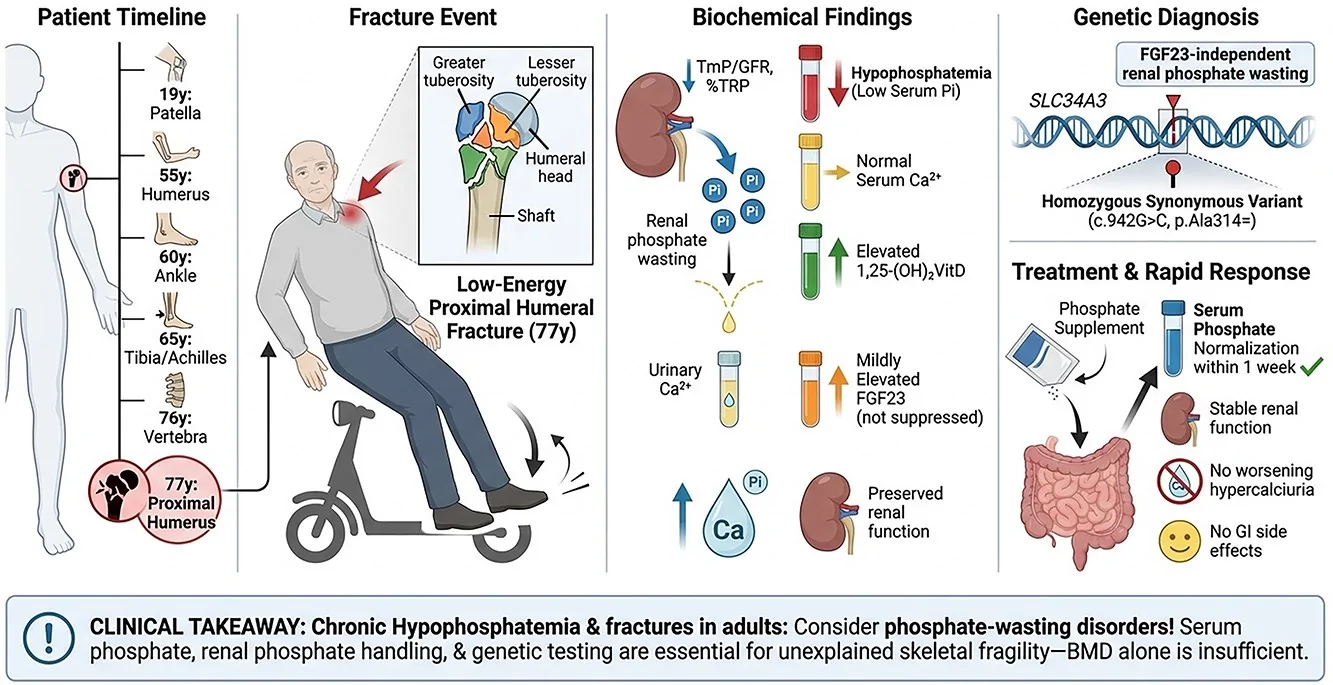

## Introduction

Persistent hypophosphatemia impairs the mineralization of newly formed osteoid, resulting in rickets in children and osteomalacia in adults. Clinical manifestations may include bone pain, muscle weakness, impaired mobility, pseudofractures, and recurrent fractures. The causes of hypophosphatemia are heterogeneous and include reduced intestinal absorption, intracellular redistribution, and renal phosphate wasting. In patients with persistent hypophosphatemia, evaluation of renal phosphate handling is essential. A reduced tubular maximum reabsorption of phosphate normalized to the glomerular filtration rate (TmP/GFR) indicates inappropriate renal phosphate wasting and facilitates further classification into FGF23-mediated and FGF23-independent disorders.^1–5^

Hereditary hypophosphatemic rickets with hypercalciuria (HHRH) is a rare autosomal recessive renal phosphate-wasting disorder caused by biallelic variants in *SLC34A3*.^6–9^  *SLC34A3* encodes NaPi-IIc, a sodium-dependent phosphate cotransporter expressed at the apical membrane of renal proximal tubular cells, where it contributes to the reabsorption of filtered phosphate.^7–10^ Impaired NaPi-IIc function reduces renal phosphate reabsorption and leads to chronic hypophosphatemia. The resulting reduction in serum phosphate stimulates 1,25(OH)2D3 production, increasing the intestinal absorption of both phosphate and calcium. Increased calcium absorption, together with normal or suppressed PTH secretion, contributes to hypercalciuria and may predispose affected patients to nephrolithiasis or nephrocalcinosis.^6–10^ Unlike FGF23-mediated hypophosphatemic disorders, HHRH is caused primarily by defective renal phosphate transport rather than excessive FGF23 activity. Oral phosphate supplementation alone is therefore generally recommended, whereas active vitamin D analogs may further increase intestinal calcium absorption and aggravate hypercalciuria.^6^^,^^8–10^

HHRH is usually recognized during childhood or early adulthood because of rickets, growth impairment, lower-limb deformity, bone pain, hypercalciuria, or nephrolithiasis.^6–10^ However, the clinical spectrum is broad, and some patients with relatively mild biochemical or skeletal manifestations remain undiagnosed until adulthood.^9^^,^^11^ In older patients, symptoms may be nonspecific, serum alkaline phosphatase (ALP) may not be markedly elevated, and reduced BMD may initially lead to the consideration of osteoporosis. Nevertheless, an appropriate evaluation for secondary causes of skeletal fragility, including the measurement of serum calcium and phosphate, remains important when an underlying metabolic bone disorder is suspected.^1–3^^,^^11^^,^^12^

We report the case of a 77-yr-old man with a late-diagnosed *SLC34A3*-related hereditary hypophosphatemic osteomalacia who presented with a low-energy proximal humeral 4-part fracture. This case illustrates that inherited renal phosphate-wasting disorders may remain unrecognized until an advanced age and emphasizes the diagnostic value of serum phosphate measurement, the assessment of renal phosphate handling, and genetic testing in patients with otherwise unexplained skeletal fragility.

## Materials and methods

### Patient and clinical evaluation

The patient was a 77-yr-old Japanese man who lived alone and had no known family history of recurrent fractures, rickets, nephrolithiasis, hypophosphatemia, or a diagnosed metabolic bone disease. He had a medical history of hypertension, dyslipidemia, and benign prostatic hyperplasia, with no history of chronic kidney disease or glucocorticoid exposure. At the time of the baseline evaluation, his regular medications were tamsulosin 0.2 mg, amlodipine 5 mg, olmesartan 20 mg, and rosuvastatin 2.5 mg, each administered orally once daily after breakfast. He had no previous exposure to osteoporosis medications, calcium or vitamin D supplements, phosphate preparations, systemic glucocorticoids, or other medications known to cause renal phosphate wasting. The patient was right-hand dominant. The diagnostic evaluation was initiated after the patient sustained a right proximal humeral fracture when he lost his balance and fell from a nearly stationary motor scooter while attempting to avoid a collision with an automobile. Given the low-energy injury mechanism and his extensive history of previous fractures and musculoskeletal injuries, a comprehensive metabolic bone evaluation was undertaken. A detailed orthopedic history was obtained through a patient interview and a review of the available medical records. For each previous fracture or musculoskeletal injury, the anatomical site, age at occurrence, reported injury mechanism, and treatment were recorded where available. The records were also reviewed to determine whether these events had previously been classified as fragility fractures or had prompted a biochemical evaluation for an underlying metabolic bone disorder. The patient was specifically questioned regarding chronic bone or musculoskeletal pain, proximal muscle weakness, gait disturbance, limitations in the activities of daily living, childhood growth impairment or lower-limb deformity, dental abnormalities, and nephrolithiasis. Written informed consent for the publication of this case report was obtained from the patient in accordance with institutional and journal requirements.

### Biochemical and urinary measurements

Baseline biochemical evaluation included measurements of serum calcium, phosphate, creatinine, ALP, intact PTH, 25OHD, and 1,25(OH)2D3 to assess vitamin D status and metabolism, and intact FGF23. Serum FGF23 was measured using a chemiluminescent enzyme immunoassay. Renal function was assessed by serum creatinine and estimated GFR (eGFR). Bone turnover markers were evaluated at baseline, including bone-specific ALP (BAP) and tartrate-resistant acid phosphatase 5b (TRACP-5b). Urinary measurements were obtained from spot urine samples and included urinary calcium, phosphate, and creatinine concentrations. Baseline biochemical and urinary parameters were assessed before the initiation of phosphate therapy. Serum calcium, phosphate, creatinine, ALP, and urinary calcium, phosphate, and creatinine were repeatedly measured during follow-up to evaluate the treatment response and the renal handling of phosphate and calcium. Intact FGF23 was measured only at baseline and was not reassessed during follow-up.

### Assessment of renal phosphate handling

Renal handling of phosphate was evaluated using the tubular reabsorption of phosphate (%TRP) and the tubular maximum reabsorption of phosphate normalized to GFR (TmP/GFR). These parameters were calculated from simultaneously obtained serum and spot urine concentrations of phosphate and creatinine using standard equations. %TRP was calculated to assess the fractional reabsorption of filtered phosphate, and TmP/GFR was derived to quantify the renal threshold for phosphate reabsorption. Baseline values were obtained prior to initiation of phosphate supplementation, and serial assessments were performed during follow-up to evaluate changes in renal phosphate handling in response to therapy.

### BMD assessment

BMD was assessed by DXA. Measurements were obtained at the LS and FN. BMD values were expressed as percentages of the young adult mean (YAM), in accordance with standard clinical practice in Japan. These measurements were performed as part of the metabolic bone evaluation to assess skeletal status at the time of diagnosis.

### Genetic analysis

Genetic analysis was performed using a targeted next-generation sequencing panel designed for hereditary hypophosphatemic disorders. The panel covered the protein-coding exons and flanking intronic regions (±10 base pairs) of genes associated with renal phosphate wasting and hypophosphatemic rickets, including *SLC34A3*. Library preparation was performed using a hybrid capture-based method, and sequencing data were analyzed by comparison with the human reference genome (GRCh38/hg38). Variant calling focused on single-nucleotide variants and small insertions or deletions with low population frequency. Variant nomenclature followed the Human Genome Variation Society guidelines using the reference transcript NM_080877.2, and zygosity was determined by sequence analysis.

### Phosphate supplementation and follow-up

Oral phosphate supplementation was initiated using Hosribon Combination Granules (Zeria Pharmaceutical Co., Ltd, Tokyo, Japan), containing monobasic sodium phosphate monohydrate and dibasic sodium phosphate anhydrous. The patient received 1200 mg of elemental phosphorus per day as 12 sachets, each providing 100 mg of elemental phosphorus, divided into 3 daily doses. This dosage was used from treatment initiation and was continued without dose adjustment during the 3-mo follow-up period. His pre-existing regular medications were continued without modification during phosphate treatment, and no additional medication with a known effect on phosphate metabolism was initiated during the follow-up period.

During follow-up, serial biochemical and urinary assessments were performed to monitor serum phosphate and calcium levels, renal function, and urinary calcium excretion. Renal phosphate handling parameters, including %TRP and TmP/GFR, were repeatedly evaluated to assess changes in phosphate reabsorption after the initiation of therapy. Safety monitoring included gastrointestinal symptoms, hypercalciuria, nephrolithiasis or nephrocalcinosis, and renal dysfunction. Treatment adherence was assessed during follow-up.

### Histopathological examination

Bone tissue obtained during surgery was submitted for histopathological examination. Specimens were fixed in formalin, decalcified, embedded in paraffin, and sectioned according to standard procedures. Sections were stained with hematoxylin and eosin and examined by conventional light microscopy, with additional evaluation under polarized light. No additional special stains, undecalcified bone preparation, tetracycline labeling, or quantitative bone histomorphometric analysis were performed.

## Results

### Proximal humeral fracture and orthopedic findings

**Figure 1:**
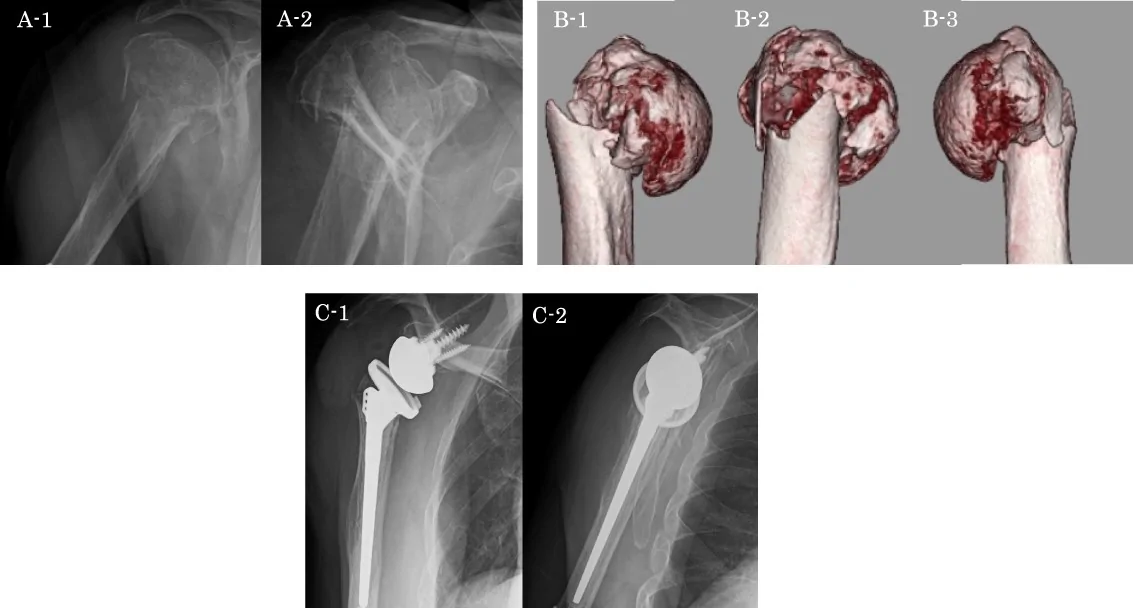
Imaging findings of the right proximal humeral fracture and postoperative outcome. (A-1) Anteroposterior radiograph of the right shoulder demonstrating a severely comminuted proximal humeral fracture. (A-2) Scapular Y-view radiograph showing displacement and comminution of the fracture fragments. (B-1) Three-dimensional CT (3D-CT) reconstruction viewed from the anterior aspect. (B-2) 3D-CT reconstruction viewed from the lateral aspect. (B-3) 3D-CT reconstruction viewed from the posterior aspect, illustrating the complex fracture morphology. (C-1) Postoperative anteroposterior radiograph following reverse shoulder arthroplasty. (C-2) Postoperative scapular Y-view radiograph demonstrating implant placement.

The patient sustained a right proximal humeral fracture while riding a motor scooter. As he attempted to avoid a collision with an automobile, he lost his balance and fell sideways from the scooter without direct contact with the automobile. At the time of the fall, the scooter was nearly stationary. He directly struck his right shoulder against the road surface. The injury did not involve a fall from a height or a high-speed impact. Initial radiographs demonstrated a severely comminuted proximal humeral fracture, which was classified as a Neer 4-part fracture (Figure 1A).^13^ Three-dimensional computed tomography further demonstrated a complex fracture morphology with marked displacement and comminution of the fracture fragments (Figure 1B). Given the fracture configuration, surgical management was selected, and a reverse shoulder arthroplasty was performed 9 d after the injury. During surgery, bone tissue samples were obtained and submitted for a histopathological examination. No intraoperative complications were reported. Postoperative radiographs demonstrated satisfactory implant positioning following the reverse shoulder arthroplasty (Figure 1C). The postoperative course was uneventful, and the patient proceeded with standard postoperative management.

### Previous fracture and musculoskeletal history

The patient had experienced multiple fractures and musculoskeletal injuries over several decades. At 19 yr of age, he sustained a patellar fracture that was treated with open reduction and internal fixation. At 55 yr, he sustained a supracondylar fracture of the humerus and underwent operative fixation. At 60 yr, he sustained a posterior malleolar fracture-dislocation of the ankle, which was treated surgically. At 65 yr, he sustained a tibial plateau fracture requiring open reduction and internal fixation, and he also underwent surgical repair of an Achilles tendon rupture. At 76 yr, he sustained an L3 vertebral fracture that was managed conservatively. All previous fractures were reported to have followed traumatic events; however, the detailed mechanisms of injury could not be confirmed from the available records. These fractures had not been formally classified as fragility fractures at the time, and no previous comprehensive evaluation for chronic hypophosphatemia, renal phosphate wasting, or a hereditary metabolic bone disease was documented. Despite this extensive orthopedic history, the patient did not recall chronic bone or musculoskeletal pain between injury episodes. He reported no subjective proximal muscle weakness, gait disturbance, or persistent limitation in mobility or in activities of daily living before the current injury. He also reported no history of childhood growth impairment, lower-limb deformity, dental abnormalities, or nephrolithiasis.

### Baseline biochemical and urinary findings

Baseline biochemical evaluation showed hypophosphatemia, with a serum phosphate level of 2.0 mg/dL (reference range, 2.7-4.6 mg/dL), while serum calcium was within the reference range at 8.9 mg/dL (reference range, 8.8-10.1 mg/dL). Renal function was preserved, with a serum creatinine level of 0.88 mg/dL (reference range, 0.65-1.07 mg/dL) and an eGFR of 64.1 mL/min/1.73 m^2^ (reference, ≥60 mL/min/1.73 m^2^). Serum ALP was 80 U/L (reference range, 38-113 U/L). Intact PTH was 48 pg/mL (reference range, 10-65 pg/mL). Assessment of vitamin D metabolism showed a serum 25-hydroxyvitamin D level of 24.8 ng/mL (reference, ≥20 ng/mL) and an elevated serum 1,25(OH)2D3 level of 88 pg/mL (reference range, 20-60 pg/mL). Serum intact fibroblast growth factor 23 measured by chemiluminescent enzyme immunoassay was mildly elevated at 34.8 pg/mL (reference, <30 pg/mL). Baseline bone turnover markers included a BAP level of 17.1 μg/L (reference range, 3.8-22.6 μg/L) and a tartrate-resistant acid phosphatase 5b level of 353 mU/dL (reference range, 170-590 mU/dL). Urinary analysis from spot urine samples demonstrated urinary calcium of 11.8 mg/dL, urinary phosphate of 25.4 mg/dL, and urinary creatinine of 56.4 mg/dL. These baseline biochemical and urinary findings are summarized in Table 1.

**Table 1:** Baseline biochemical, urinary, and renal phosphate handling findings at diagnosis.

**Parameter**	**Value**	**Reference range**
**Calcium (mg/dL)**	8.9	8.8-10.1
**Phosphate (mg/dL)**	2.0	2.7-4.6
**Creatinine (mg/dL)**	0.88	0.65-1.07
**eGFR (mL/min/1.73 m** ^**2**^ **)**	64.1	≥60
**ALP (U/L)**	80	38-113
**Intact PTH (pg/mL)**	48	10-65
**25OHD (ng/mL)**	24.8	≥20
**1,25(OH)2D3 (pg/mL)**	88	20-60
**Intact FGF23 (pg/mL)**	34.8	<30^a^
**BAP (μg/L)**	17.1	3.8-22.6
**TRACP-5b (mU/dL)**	353	170-590
**Urinary calcium (mg/dL)**	11.8	Not established
**Urinary phosphate (mg/dL)**	25.4	Not established
**Urinary creatinine (mg/dL)**	56.4	Not established
**%TRP (%)**	80.2	>85
**TmP/GFR (mg/dL)**	1.2	2.3-4.3
**LS BMD (%YAM)**	92	—
**LS T-score**	−0.7	—
**FN BMD (%YAM)**	66	—
**FN T-score**	−2.9	—

Abbreviations: ALP, alkaline phosphatase; BAP, bone-specific ALP; eGFR, estimated glomerular filtration rate; FGF23, fibroblast growth factor 23; %TRP, tubular reabsorption of phosphate; TmP/GFR, tubular maximum phosphate reabsorption per GFR; YAM, young adult mean; tartrate-resistant acid phosphatase 5b (TRACP-5b).

a Reference range according to the assay used in this study.

### Renal phosphate handling at baseline

Evaluation of renal phosphate handling at baseline demonstrated the %TRP of 80.2%, below the expected value of >85%. The tubular maximum reabsorption of phosphate normalized to GFR (TmP/GFR) was reduced to 1.2 mg/dL (reference range, 2.3-4.3 mg/dL), indicating inappropriate renal phosphate wasting. These parameters were calculated using simultaneously obtained serum and spot urine measurements. Baseline renal phosphate handling parameters are summarized in Table 1.

### BMD

BMD assessed by DXA demonstrated a LS value corresponding to 92% of the YAM (T-score, −0.7). At the FN, BMD was reduced to 66% of the YAM (T-score, −2.9). These measurements were obtained during the diagnostic evaluation.

### Genetic findings

Genetic analysis identified a homozygous synonymous variant in *SLC34A3*, c.942G > C (p.Ala314=), based on the reference transcript NM_080877.2. No other pathogenic or likely pathogenic variants were detected in the genes included in the targeted panel for hereditary hypophosphatemic disorders. The patient was unmarried and had no children or grandchildren. His parents were deceased or of advanced age, and no other family members were available for biochemical or genetic testing. Therefore, a segregation analysis was not possible.

### Response to oral phosphate supplementation

After the initiation of oral phosphate supplementation, serum phosphate levels increased rapidly and normalized within 1 wk. Serum phosphate rose from 2.0 mg/dL at baseline to 4.0 mg/dL after 1 wk and remained stable thereafter, measuring 4.0 mg/dL at 4 wk and 3.9 mg/dL at 3 mo. Serum calcium levels remained within or slightly below the reference range throughout the follow-up (8.4-8.9 mg/dL). Renal function remained preserved during treatment, with stable serum creatinine levels (0.88 mg/dL at baseline, 0.69 mg/dL at 1 wk, 0.76 mg/dL at 4 wk, and 0.78 mg/dL at 3 mo) and no evidence of treatment-related renal impairment. Serial measurements demonstrated changes in renal phosphate handling following therapy. The %TRP decreased from 80.2% at baseline to approximately 69.9% at wk 1 and remained relatively stable thereafter (74.6% at wk 4 and 64.9% at 3 mo). In contrast, the tubular maximum reabsorption of phosphate normalized to GFR (TmP/GFR) increased from 1.2 mg/dL at baseline to approximately 1.9 mg/dL at wk 1, 2.1 mg/dL at wk 4, and 2.5 mg/dL at 3 mo. These post-treatment values were interpreted in the context of ongoing phosphate supplementation and increased serum and urinary phosphate concentrations. Serum ALP levels increased transiently during follow-up, rising from 80 U/L at baseline to 121 U/L at wk 2, followed by a gradual decline to 89 U/L at wk 4 and 97 U/L at 3 mo. Urinary phosphate excretion increased after treatment initiation, reflecting phosphate supplementation, whereas urinary calcium excretion showed an overall decline during follow-up. No gastrointestinal adverse effects, including diarrhea, abdominal pain, or nausea, were reported. No worsening of hypercalciuria, nephrolithiasis, nephrocalcinosis, or renal dysfunction was observed during the 3-mo follow-up period. The patient continued treatment without interruption, and no adherence problems were identified. Intact FGF23 was not remeasured after initiation of phosphate supplementation. Serial biochemical, urinary, and renal phosphate handling data, together with the corresponding reference ranges, are summarized in Table 2.

**Table 2:** Serial biochemical, urinary, and renal phosphate handling changes after initiation of oral phosphate supplementation.

**Parameter**	**Baseline**	**1 wk**	**2 wk**	**4 wk**	**3 mo**	**Reference range**
**Calcium (mg/dL)**	8.9	8.4	8.7	8.7	8.9	8.8-10.1
**Phosphate (mg/dL)**	2.0	4.0	3.8	4.0	3.9	2.7-4.6
**Creatinine (mg/dL)**	0.88	0.69	—	0.76	0.78	0.65-1.07
**eGFR (mL/min/1.73 m** ^**2**^ **)**	64.1	—	—	—	73.2	≥60
**ALP (U/L)**	80	—	121	89	97	38-113
**Urinary calcium (mg/dL)**	11.8	10.9	—	11.9	3.6	Not established
**Urinary phosphate (mg/dL)**	25.4	97.2	—	173.1	35.8	Not established
**Urinary creatinine (mg/dL)**	56.4	—	—	122.8	21	Not established
**%TRP (%)**	80.2	69.9	—	74.6	64.9	>85
**TmP/GFR (mg/dL)**	1.2	1.9	—	2.1	2.5	2.3-4.3

Abbreviations: eGFR, estimated glomerular filtration rate; %TRP, tubular reabsorption of phosphate; TmP/GFR, tubular maximum phosphate reabsorption per GFR.

Oral phosphate supplementation consisted of Hosribon Combination Granules providing 1200 mg of elemental phosphorus per day, divided into 3 daily doses. Intact FGF23 was measured only at baseline and was not reassessed during follow-up.

A dash indicates that the parameter was not measured or was unavailable at the corresponding time point.

### Histopathological findings

**Figure 2:**
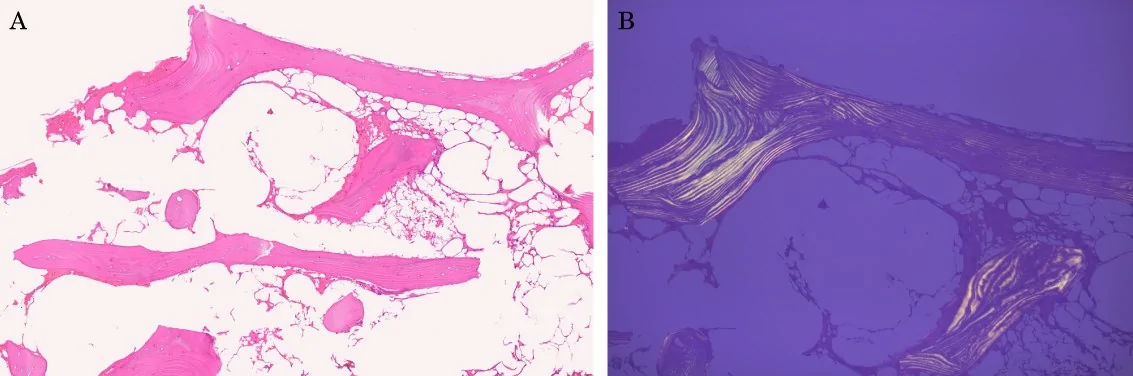
Histopathological findings of bone tissue obtained during surgery. (A) Hematoxylin and eosin-stained decalcified section showing trabecular bone with preserved lamellar architecture and marrow spaces. No malignant features or other specific pathological bone lesions are identified. (B) Polarized light microscopy demonstrating organized lamellar bone architecture. These decalcified specimens were not suitable for quantitative assessment of osteoid volume or mineralization.

Histopathological examination of the surgically obtained bone tissue was performed using hematoxylin and eosin-stained, decalcified paraffin sections. The sections demonstrated trabecular bone with a preserved lamellar architecture, and no malignant or other specific pathological bone lesion was identified. Polarized light microscopy demonstrated an organized lamellar bone architecture. No conspicuous accumulation of unmineralized osteoid was identified in the examined sections; however, the decalcified specimens were not suitable for a definitive assessment of mineralization defects or a quantitative diagnosis of osteomalacia. Representative histopathological findings are shown in Figure 2.

## Discussion

This case illustrates a very late-diagnosed presentation of hereditary hypophosphatemic osteomalacia with hypercalciuria in a 77-yr-old man. The principal findings were persistent renal phosphate wasting despite preserved renal function, elevated 1,25(OH)2D3, hypercalciuria, the identification of a homozygous synonymous *SLC34A3* variant, and a rapid biochemical response to oral phosphate supplementation without active vitamin D analogs. The diagnosis was prompted by the occurrence of a proximal humeral fracture after low-energy trauma in the context of a long history of previous fractures. These findings broaden the recognized clinical spectrum of HHRH and demonstrate that inherited phosphate-wasting disorders may remain unrecognized until an advanced age.

HHRH is caused by impaired renal phosphate reabsorption due to the dysfunction of NaPi-IIc, the sodium-dependent phosphate cotransporter encoded by *SLC34A3*. NaPi-IIc is expressed at the apical membrane of proximal tubular cells and contributes to the reclamation of filtered phosphate from the renal tubular lumen. Reduced NaPi-IIc activity lowers the renal threshold for phosphate reabsorption, resulting in persistent urinary phosphate wasting and hypophosphatemia. In response to reduced serum phosphate, renal production of 1,25(OH)2D3 increases, thereby enhancing the intestinal absorption of both phosphate and calcium. Although increased intestinal phosphate absorption partially compensates for renal phosphate loss, increased calcium absorption leads to hypercalciuria and may predispose affected patients to nephrolithiasis or nephrocalcinosis.^6–10^ This biochemical sequence was reflected in the present patient by hypophosphatemia, a reduced TmP/GFR, elevated 1,25(OH)2D3, and increased urinary calcium excretion. The relevance of NaPi-IIc dysfunction in the present case is further supported by the combination of a reduced TmP/GFR and persistent urinary phosphate loss despite preserved renal function. These findings indicate an inappropriately low renal threshold for phosphate reabsorption and support impaired NaPi-IIc-mediated phosphate transport as the principal mechanism of phosphate wasting.

The therapeutic approach to HHRH differs fundamentally from that used for FGF23-mediated hypophosphatemic disorders. In FGF23-mediated disease, excessive or inappropriately normal FGF23 activity simultaneously reduces proximal tubular phosphate reabsorption and suppresses 1,25(OH)2D3 production. Conventional treatment therefore often requires both phosphate and active vitamin D supplementation, and anti-FGF23 therapy may be appropriate in selected disorders. In HHRH, however, the primary defect is intrinsic loss of renal phosphate transport through NaPi-IIc, while endogenous 1,25(OH)2D3 production is already increased. Oral phosphate supplementation addresses the principal biochemical deficit and may also reduce the stimulus for excessive 1,25(OH)2D3 production. The routine addition of active vitamin D is generally unnecessary and may further increase intestinal calcium absorption, thereby worsening hypercalciuria and the risk of nephrocalcinosis.^3^^,^^6^^,^^8–10^ The rapid normalization of serum phosphate without deterioration in renal function or a sustained worsening of urinary calcium in the present case was consistent with this treatment principle.

A notable finding in this patient was that the intact FGF23 was mildly above the assay reference limit and was not appropriately suppressed despite hypophosphatemia. This finding is not typical of classical HHRH, in which FGF23 is generally low or appropriately suppressed because the primary defect is an FGF23-independent renal phosphate wasting. Several factors may have contributed to this result, including biological variability, assay-related variation, the timing of sampling, or an additional influence on FGF23 regulation. However, serial FGF23 measurements were not obtained, and it therefore remains unclear whether the baseline elevation was persistent or whether FGF23 changed in response to phosphate supplementation. Despite this atypical finding, the overall biochemical phenotype—including a reduced TmP/GFR, elevated 1,25(OH)2D3, hypercalciuria, and the response to phosphate supplementation without active vitamin D—was more consistent with an FGF23-independent renal phosphate-wasting disorder than with a classical FGF23-mediated condition. Nevertheless, the mildly elevated FGF23 level, together with the synonymous nature of the identified *SLC34A3* variant and the absence of functional or segregation studies, warrants cautious interpretation of the molecular diagnosis.

A key feature of this case is the exceptionally late recognition of the phosphate-wasting disorder. The mechanism underlying the attenuated and delayed presentation remains uncertain, although a relatively mild defect in renal phosphate transport is one possible explanation. Classic HHRH caused by biallelic loss-of-function variants in *SLC34A3* typically presents in childhood with overt rickets, growth retardation, or nephrolithiasis, accompanied by marked biochemical abnormalities, including severe hypophosphatemia, elevated 1,25(OH)2D3 levels, and hypercalciuria.^10^ In contrast, recent reviews have emphasized that milder defects in renal phosphate transport may result in attenuated biochemical phenotypes and delayed clinical presentation, particularly in adults, in whom hypophosphatemia may be overlooked or attributed to age-related skeletal changes.^2^^,^^14^ In the present case, despite lifelong renal phosphate wasting, serum ALP levels were within the reference range at diagnosis, and classical manifestations such as bone pain, rickets, or nephrolithiasis were absent. The absence of overt childhood rickets suggests a clinically attenuated phenotype; however, the underlying molecular mechanism cannot be determined from the available data. Although the identified *SLC34A3* variant was synonymous, such variants have been recognized as potentially pathogenic through effects on mRNA stability, splicing efficiency, or translational regulation, particularly when present in the homozygous state and accompanied by a characteristic biochemical phenotype.^2^ While functional studies were not performed, the coexistence of a homozygous *SLC34A3* variant and a biochemical phenotype largely consistent with HHRH supports a *SLC34A3*-related phosphate-wasting disease; however, the synonymous nature of the variant, the mildly elevated FGF23 level, and the absence of functional and segregation studies preclude definitive molecular confirmation. Together, these observations suggest that relatively mild phosphate-wasting phenotypes may delay recognition until an advanced age.

The absence of a known family history does not exclude HHRH because the disorder is inherited in an autosomal recessive manner, and affected individuals may be born to clinically unaffected heterozygous parents. In the present case, biochemical testing and segregation analysis were not performed in family members, and the inheritance pattern of the identified variant within the family could therefore not be confirmed. Heterozygous *SLC34A3* variants may also be associated with variable biochemical manifestations, including mild hypophosphatemia, hypercalciuria, nephrolithiasis, or a reduced BMD.^8–10^ The patient was unmarried and had no children or grandchildren. His parents were deceased or of advanced age, and no relatives were available for biochemical or genetic evaluation. Therefore, a segregation analysis and an assessment of potential carrier phenotypes within the family could not be performed. In similar cases, genetic counseling and a targeted biochemical or genetic evaluation of available first-degree relatives may be considered.

Another important clinical implication of this case is the need to consider secondary causes of skeletal fragility when the fracture history, clinical presentation, or biochemical findings are not fully explained by primary osteoporosis. Clinical guidelines recommend an evaluation for secondary contributors to skeletal fragility, with additional investigations guided by the clinical context.^1–4^^,^^11^^,^^12^ Hypophosphatemia may be overlooked because serum phosphate is not included in all routine biochemical panels, and disorders of phosphate metabolism may present with nonspecific or subtle musculoskeletal manifestations.^14^ Adult fractures are common in older populations, but recurrent fractures should still prompt a consideration of secondary skeletal disorders when the overall clinical context is not fully explained by primary osteoporosis.^15^ In the present patient, the proximal humeral fracture occurred after a low-energy fall in the context of an extensive history of previous fractures and musculoskeletal injuries. Although the fracture configuration itself was not diagnostic of a metabolic bone disorder, the low-energy mechanism and longitudinal fracture history prompted a broader biochemical evaluation. Subsequent biochemical evaluation demonstrated hypophosphatemia and inappropriate renal phosphate wasting, which provided the decisive evidence for further investigation. Similar to other reported forms of osteomalacia, fractures may precede the recognition of the underlying biochemical abnormality, particularly when characteristic symptoms are absent.^16^ These observations support an integrated diagnostic approach combining fracture history, injury mechanism, BMD, serum calcium and phosphate measurements, and the assessment of renal phosphate handling.

This case underscores several clinically important considerations for the evaluation of fractures in older adults. First, it highlights the persistent under-recognition of hypophosphatemia as a contributor to skeletal fragility, largely because serum phosphate is not routinely included in standard biochemical panels and its clinical relevance is often underestimated in adult patients.^2^^,^^12^^,^^17^ As emphasized in recent expert guidance and large cohort analyses, chronic hypophosphatemia—whether FGF23-mediated or FGF23-independent—can present predominantly with fractures and musculoskeletal symptoms, particularly in adults, without overt biochemical abnormalities such as elevated ALP or severe bone pain.^11^^,^^18^ Second, this case illustrates the limitations of diagnostic strategies that rely primarily on BMD measurements. Although DXA is widely used to assess fracture risk, it may fail to capture disorders of bone quality, such as osteomalacia, in which impaired mineralization rather than reduced bone mass is the dominant pathological feature. Reduced BMD alone does not distinguish primary osteoporosis from osteomalacia. This distinction is clinically important because antiresorptive therapy addresses excessive bone resorption but does not correct defective mineralization caused by chronic hypophosphatemia. Initiating antiresorptive treatment without first identifying and correcting the underlying phosphate-wasting disorder may delay disease-specific therapy and complicate the interpretation of subsequent skeletal outcomes. Therefore, serum calcium and phosphate, along with other appropriate tests for secondary causes of skeletal fragility, should be assessed before initiating osteoporosis-specific pharmacotherapy when the clinical or biochemical presentation raises concern for osteomalacia.^1–4^^,^^11^^,^^12^ Expert consensus statements on hypophosphatemic disorders consistently emphasize the importance of assessing renal phosphate handling, including %TRP and TmP/GFR, in patients with unexplained fractures or recurrent skeletal injuries, regardless of age.^17^^,^^18^ Finally, recognition of HHRH has direct therapeutic implications because treatment should target renal phosphate wasting while avoiding unnecessary active vitamin D supplementation that may aggravate hypercalciuria. Together, these considerations support a diagnostic approach that integrates injury mechanism and longitudinal fracture history with targeted biochemical evaluation. Increased awareness of phosphate metabolism and closer collaboration between orthopedic surgeons and metabolic bone specialists may therefore improve diagnostic accuracy and patient outcomes, particularly in elderly individuals presenting with recurrent or otherwise unexplained skeletal fragility. This perspective is consistent with emerging frameworks that conceptualize bone as an active component of whole-body endocrine and metabolic regulation, in which disturbances of mineral metabolism may initially manifest through orthopedic events rather than classic endocrine symptoms.^19^

Several limitations of this report should be acknowledged. First, this is a single-case observation, and the findings may not be generalizable to all patients with HHRH or hypophosphatemic disorders. Second, although genetic analysis identified a homozygous synonymous *SLC34A3* variant with strong phenotype-genotype concordance, functional studies were not performed to assess its impact on NaPi-IIc expression or activity directly. In addition, a detailed biochemical evaluation of family members and a segregation analysis were not available, limiting the confirmation of the inheritance pattern and the assessment of potential carrier phenotypes. Third, histopathological assessment was limited to decalcified paraffin-embedded specimens stained with hematoxylin and eosin and examined under polarized light. No special stains, undecalcified bone preparation, tetracycline labeling, or dynamic bone histomorphometry were performed. Therefore, the pathological specimens could exclude malignant or other specific bone lesions but could not definitively establish or exclude osteomalacia. Finally, serial FGF23 measurements were not performed, precluding an assessment of whether the mildly elevated baseline value was persistent or changed during phosphate supplementation. Despite these limitations, this case provides clinically meaningful insights by demonstrating that HHRH can remain unrecognized until an advanced age and may present primarily with fractures rather than classic symptoms. The diagnostic evaluation was initiated after the low-energy fracture prompted reassessment of the patient’s extensive fracture history and biochemical evaluation for secondary causes of skeletal fragility. This report illustrates that HHRH may remain unrecognized until an advanced age and should be considered in patients with recurrent fractures or skeletal fragility that is not fully explained by BMD. Appropriate evaluation of serum phosphate and, when indicated, renal phosphate handling before the initiation of osteoporosis-specific therapy may facilitate an earlier diagnosis and disease-specific treatment.

## Data Availability

The data supporting the findings of this case report are available from the corresponding author upon reasonable request, where permitted by applicable ethical and privacy considerations.
